# Illustration d’une spondyloptose dégénérative du sujet jeune

**DOI:** 10.11604/pamj.2017.26.183.11509

**Published:** 2017-03-29

**Authors:** Ababacar Abdoulaye Traore, Badreeddine Alami

**Affiliations:** 1 Faculté de Médecine et de Pharmacie, Université Sidi Mohamed Ben Abdellah, Service de Radiologie du CHU Hassan II Fès, Fès, Maroc

**Keywords:** Spondyloptose dégénérative, patient jeune, tomodensitométrie

## Image en médecine

Il s’agit d’un jeune patient de 17 ans, sans facteurs de risques congénitaux ou héréditaires ni de notion de traumatisme ayant consulté pour une douleur lombaire avec troubles importants de la statique. A l’examen physique, les régions glutéales étaient aplaties et le bord supérieur de la première vertèbre sacrée était palpable dans le creux lombaire. Le patient présentait par ailleurs une hanche abaissée et des déhanchements à la marche. Une tomodensitométrie du rachis dorso-lombo-sacrée, demandée pour bilan étiologique, a mis en évidence un glissement complet vers l’avant du corps vertébral de L5, dont le plateau vertébral inferieur surplombait le bord antéro supérieur de la première vertèbre sacrée avec un aspect verticalisé du sacrum. Il s’y associe une lyse isthmique gauche de L5 et des plages ostéo condensantes réactionnelles des berges osseuses. Une spondyloptose dégénérative par lyse isthmique a été porté comme diagnostic. En effet, elle constitue le grade V d’évolution d’une spondylolisthésis. En effet, la spondyloptose est une dislocation vertébrale complète de la cinquième vertèbre lombaire (L5) venant se placer en avant du sacrum (S1). Elle correspond au dernier stade d’un spondylolisthésis. La spondylolisthésis serait classée en cinq types: dysplasique, isthmique, dégénératif, traumatique et pathologique. Le traitement d’une spondyloptose efficace est chirurgical chez les adultes, la thérapie par réduction reste une alternative chez les enfants. Notre patient a été mis sous traitement conservateur antalgique avec immobilisation simple, vu l’absence d’une issue chirurgicale.

**Figure 1:**
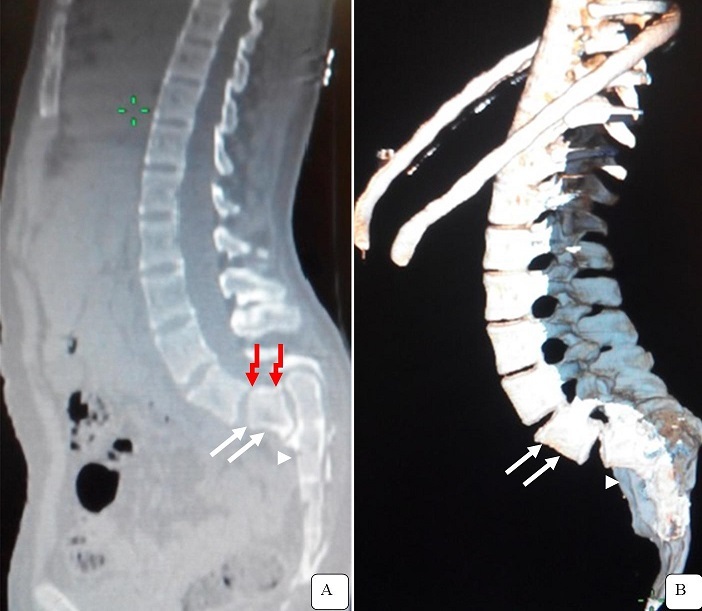
Coupes tomodensitométriques en reconstruction sagittale (A) et 3D VR (B) objectivent une dislocation complète du corps vertébral de L5 en avant du sacrum (flèche blanche); noter l’aspect verticalisé du sacrum (tête de flèche)

